# Enteral plasma feeding improves gut function and immunity in piglets after birth asphyxia

**DOI:** 10.1038/s41390-024-03376-0

**Published:** 2024-07-21

**Authors:** Mads Jacob Bagi Nordsten, Christina L. Winther, Maria Mathilde Haugaard, Kerstin Skovgaard, Thomas Thymann, Per T. Sangild

**Affiliations:** 1 Comparative Pediatrics and Nutrition, Department of Veterinary and Animal Sciences, Faculty of Health and Medical Sciences, University of Copenhagen, Copenhagen, Denmark; 2 Department of Pediatrics and Adolescent Medicine, Rigshospitalet, Copenhagen, Denmark; 3 Department of Biotechnology and Biomedicine, Technical University of Denmark, Lyngby, Denmark; 4 Department of Pediatrics, Odense University Hospital, Odense, Denmark; 5 Faculty of Theology, University of Copenhagen, Copenhagen, Denmark

## Abstract

**Introduction:**

Birth asphyxia may negatively affect gut function and immunity in newborns. Conversely, immunomodulatory milk diets may protect the gut and immune system against damage caused by asphyxia. Using caesarean-derived pigs as models, we hypothesised that enteral feeding with plasma improves gut and immune functions in asphyxiated newborns.

**Methods:**

Near-term pig fetuses (98% gestation,) were delivered by caesarean section after 8 min umbilical cord occlusion, leading to transient birth asphyxia (ASP, *n* = 75) and compared with non-occluded controls (CON, *n* = 69). Piglets were further randomised to supplementation with/without porcine plasma (plasma, PLA/vehicle, VEH), into bovine colostrum (first 24 h) or formula (until 72 h).

**Results:**

Compared with CON, ASP piglets took longer to achieve stable respiration and showed reduced blood pH, weight gain and survival. Independent of asphyxia, plasma supplementation reduced gut haemorrhagic lesions, permeability and inflammatory cytokines together with improved villous morphology and brush-border enzyme activities. Asphyxia reduced blood cytokine responses to ex vivo bacterial stimulation, whereas plasma supplementation ameliorated this effect.

**Conclusion:**

Dietary plasma supplementation improves survival, gut functions and immunity in both normal and asphyxiated newborns. The components in plasma that mediate gut-protective effects in piglets remain to be identified, but may benefit also birth-compromised newborn infants.

**Impact:**

- Complicated deliveries leading to birth asphyxia, may negatively affect gut, liver and immune adaptation in the first days after birth.
- Using a model of birth asphyxia in caesarean-derived piglets, we show that enteral feeding with maternal plasma exerts gut maturational and immunomodulatory effects in both control and asphyxiated animals in the first days of life.
- The mechanisms behind the gut-protective effects of plasma are unknown, but plasma components hold potential for new oral therapies for compromised newborn infants as well as piglets.

## Introduction

Birth complications, including full umbilical cord occlusion or temporary cord compressions from uterine contractions, increase the risk of neonatal cardiovascular instability and stillbirth.^1^ Impaired umbilical cord circulation during delivery may cause decreased circulatory oxygenation and pH at birth,^2^ resulting in foetal distress and asphyxia as indicated by meconium-stained amniotic fluid.^3^ In full-term infants, asphyxia-related oxygen deficits at birth reduce mesenteric blood flow^4^ affecting intestinal tissue perfusion. In neonatal piglets, acidosis-hypoxia hinders intestinal blood flow likely leading to intestinal ischaemia-reperfusion lesions,^5,6^ impaired intestinal integrity and risk of bacterial translocation and systemic infection.^7–9^ Pro-inflammatory ischaemic responses, as observed in infant^10^ and rodent studies,^11^ may also lead to liver pathology, coagulopathy and elevated transaminase levels. While clinical consequences of severe asphyxia are well documented, particularly for the developing brain, it is unclear if more moderate or transient asphyxia during delivery may affect intestinal, immune and hepatic functions.

In compromised preterm infants, the immature intestine is highly sensitive to enteral feeding, especially with formula, while mother’s own milk or colostrum generally protects against gut and immune disorders.^12,13^ Providing small amounts of maternal colostrum just after birth reduces intestinal inflammation and late-onset sepsis in preterm infants.^14^ Similarly, porcine colostrum has profound protective effects on neonatal piglets, in part due to its content of species-specific immunoglobulins (Igs) that are absorbed and systemically available in piglets.^15^ However, mammalian colostrum also contains non-species-specific bioactive components, and bovine colostrum has been shown to induce gut-protective effects in both preterm and term piglets.^16,17^ Cross-species effects of colostrum in infants could be possible via Igs working locally in the gut environment,^18,19^ while direct systemic effects are more questionable due to lack of Ig absorption and species-specific systemic immune effects of Igs.^20^ In infants, dietary IgG or IgA supplements did not consistently protect preterm infants against necrotising enterocolitis (NEC).^21,22^ Potentially, other factors than Igs contribute to the gut-protective effects of colostrum (e.g. albumin, growth factors, hormones, cytokines, immunomodulatory proteins). Since many of these bioactive compounds are highly sensitive to milk processing required for donor milk or formula production,^23,24^ dietary supplementation of such protective factors may support gut and organ development in newborns lacking access to their own mother’s milk.

Many of the bioactive components present in colostrum are also present in plasma because colostrum is a systemic extract from galactopoietic cells in the mammary gland.^16^ Feeding piglets with dried plasma during the weaning period is known to prevent intestinal infections and post-weaning diarrhoea, but the biologically active components remain elusive.^25,26^ During the first 12–24 h, newborn piglets are entirely dependent on passive immunity obtained through the absorption of Igs from colostrum, yet under conditions where maternal colostrum is not available, other milk formulations with similar biological components are required. To this end, previous research has shown that a formula supplemented with concentrated porcine plasma may improve gut maturation in newborn pigs.^27^ Newborn piglets in particular allow bioactive proteins to escape digestion in the low-proteolytic gut environment and be available for absorption by endocytosis.^28^ Hence, these bioactive proteins may act locally in the gut to bind pathogens^29^ and strengthen intestinal barrier function at a time when pigs are highly sensitive to microbial colonisation. Therefore, both preterm and term piglets have been used to model the high sensitivity of preterm infants toward NEC and feeding intolerance.^30^ In newborn piglets, unprocessed bovine colostrum is superior to infant formula in protecting the intestines,^16^ yet bovine colostrum remains inferior to porcine colostrum and milk.^31,32^ In preterm infants, supplements of bovine colostrum improved gut function but only in infants largely devoid of mother’s own milk.^33,34^

Given the content of plasma-derived compounds in colostrum, the deficiencies in formula and colostrum/milk from other species in protecting the newborn could potentially be compensated by adding species-specific plasma. On this background, we hypothesised that piglets fed bovine colostrum and formula supplemented with porcine plasma would show improved protection of their immature intestines in the immediate neonatal period. We also hypothesised that the effects would be most pronounced in piglets suffering from birth asphyxia. Following near-term caesarean section, we investigated liver function, gut structure, absorptive function and immunology in control and asphyxiated piglets. Documentation of effects of plasma-containing milk diets may pave the way for plasma-derived supplements that protect the gut and other organs after birth complications in both piglets and infants.

## Materials and methods

### Animals and interventions

**Fig. 1:**
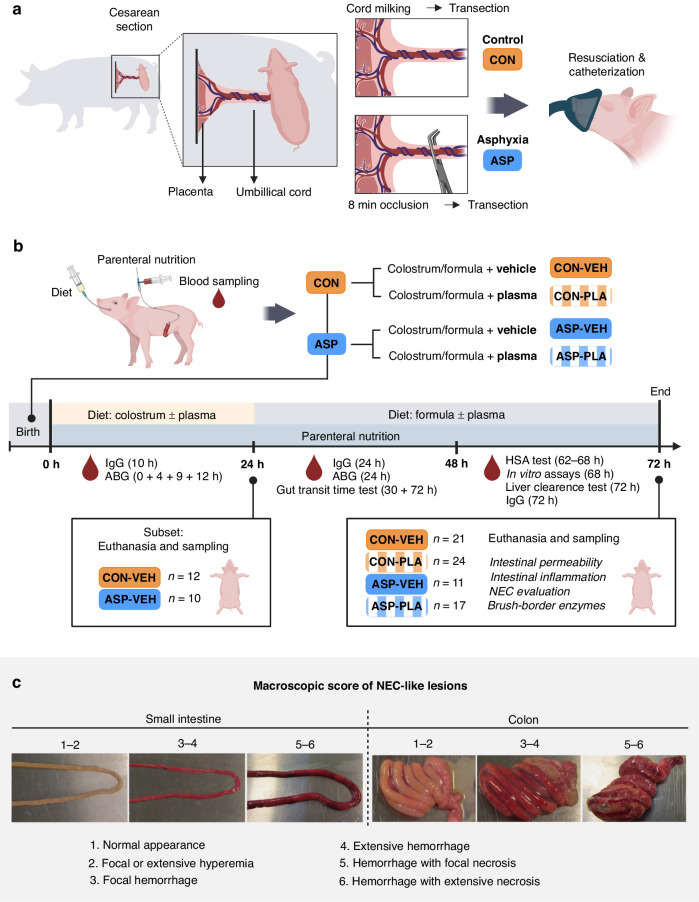
Study design and scores of different necrotizing-like lesions. Graphical illustration of experimentally induced birth asphyxia, and postnatal resuscitation (**a**). Postnatal allocation to diet types using a 2 × 2 factorial design, and blood sampling time points for measurement of IgG, human serum albumin (HSA) and arterial blood gas values (ABG), as well as ex vivo tests and liver clearance test (**b**). Examples of NEC-like lesions used for scoring at 72 h (**c**).

Experiments were conducted under an animal experimentation license (no. 2020-15-0201-00520) obtained from the Danish Animal Experiments Inspectorate. The animals were simultaneously used to study brain-related endpoints.^35^ In total 144 piglets (Large White × Danish Landrace × Duroc) were delivered by caesarean section from five sows at gestational day 113 (i.e. near-term with anticipated full term at day 117–118), as previously described.^36^ Piglets were delivered with induced birth asphyxia (ASP) or without birth asphyxia (CON). To induce asphyxia, the umbilical cord was exteriorised through a small uterine incision and completely obstructed with a naval clamp for 7.7 ± 0.7 min (mean ± SD) with the piglets remaining in utero (Fig. 1). Initially, seven CON piglets were delivered followed by seven ASP piglets. Remaining piglets in each litter were delivered alternating between CON and ASP. Based on preliminary experiments, a higher risk of resuscitation failure for ASP piglets was expected. Arterial cord blood was collected into a heparinized syringe before and after cord obstruction for ASP piglets and immediately after cord transection in CON piglets for measurements of arterial blood gases (ABL90 FlexPlus, Radiometer, Brønshøj, Denmark). Subsequent blood samples were collected at 4, 9, 12 and 24 h after delivery from an arterial catheter (Fig. 1, see more details below). Immediately after delivery, piglets received an intramuscular injection of 0.1 mL of doxapram hydrochloride and 0.1 mL of flumazenil to prevent respiratory depression induced by general anaesthesia. Piglets not breathing shortly after birth were resuscitated by non-invasive resuscitation using Lullaby Resus Plus (GE, United Kingdom), and time to reach stable respiration was recorded. Once respiration was stable, piglets were fitted with an orogastric feeding tube (6 F, Portex, Kent, UK) and an umbilical arterial catheter (4F, Portex) as previously described.^30^ Based on sex, birth weight and birth mode (CON, ASP), piglets were block randomised to two diet groups consisting of either a plasma supplementation group or a vehicle group as described in detail below. Piglets were housed individually in closed incubators and reared for either 24 h (derived from one litter *n* = 12 CON and *n* = 18 ASP piglets) or 72 h (derived from four litters, *n* = 57 CON and *n* = 57 ASP piglets). See Supplementary Table S1 for an overview of piglets allocated to each delivery group, subsequent block randomisation and reasons for euthanasia prior to planned tissue collection time.

To support fluid homoeostasis and nutrition, pigs received infusions of parenteral nutrition via the umbilical catheter (2 mL/kg/h; Kabiven, Fresenius-Kabi, Uppsala, Sweden) throughout the study. The piglets were then block randomised to receive enteral nutrition with bovine milk powders dissolved in either porcine plasma, i.e. maternal plasma collected at the time of caesarean section (plasma group, PLA) or water (vehicle group, VEH). To ensure that plasma effects could be attributed entirely to the enteral route of administration, we did not provide any intravenous infusion of maternal plasma. For enteral nutrition the first 24 h, bovine colostrum powder was dissolved at in either water or in plasma collected from the sow as described in detail below. Likewise, from 24 to 72 h, pigs were fed formula diets dissolved in either water or plasma. Details for both diets are provided in Supplementary Tables S2 and S3. To balance the dietary protein levels between PLA and VEH, we added 70 g whey protein per liter in the VEH group. The maternal plasma was collected from each sow immediately after delivery of the last foetus, via catheterisation of a large uterine artery. The blood was collected into sterile heparinized bottles (5000 IU pr. L blood), and placed at 4 °C to sediment and then centrifuged at 2000 × *g* for 20 min at 4 °C. Plasma was aliquoted into separate plastic containers and stored at 4 °C before being mixed with colostrum powder or formula ingredients. Bolus feeding started 3–5 h postdelivery and continued at 15 ml/kg/3 h until tissue collection.

### In vivo gut transit time, permeability and macromolecule absorption

Gut transit time, permeability and macromolecule absorption were assessed through various in vivo tests. For gut transit time, animals were fed an enteral formula bolus (15 mL/kg) containing 6 g/l Cr_2_O_3_ (a green dye) on day 2 at ~0 h of life. The time for the first passage of dark-green colored stool was recorded gut transit time. Intestinal permeability was assessed by feeding 15 mL/kg bolus of a 5% mannitol-lactulose solution 3 h before tissue collection. A urine sample was collected at tissue collection, and the urinary concentrations of mannitol and lactulose were measured^37^ and the lactulose/mannitol-ratio was used as a marker of intestinal permeability. To evaluate intestinal endocytosis and immunoglobulin absorption, plasma IgG concentrations were measured at 10, 24 and 72 h, as previously described.^38^ At 62 h of life, pigs were fed a 15 mL/kg bolus of the formula mixed with human serum albumin (HSA, 50 mg/mL, Sigma-Aldrich, Saint Louis, Missouri) to evaluate the absorption of macromolecules to the blood stream. Blood was sampled exactly 6 h later and analysed as previously described.^27^ Animals were prematurely euthanized if: (1) pain relief could not be achieved, (2) respiratory distress with no response to respiratory support, (3) severe diarrhoea and dehydration with no response to fluid support, (4) suspected NEC diagnosis with no response to feeding cessation, or (5) clinical signs of sepsis or blood pH < 7.1.

### Euthanasia, tissue sampling and macroscopic evaluation of NEC-like lesions

Animals were anaesthetised with an intramuscular injection of 0.1 mL/kg mixture of tiletamine, zolazepam, ketamine, xylazin and butorphanol. Once anaesthetised, blood was collected for biochemical analyses through cardiac puncture before a lethal cardiac injection of pentobarbital. Tissue from the small and large intestine were snap frozen in liquid nitrogen and stored at −80 °C, or immersion fixed in 4% paraformaldehyde followed by paraffin embedding. For the stomach, small intestine and colon, a six-grade scoring system^39^ was used to evaluate macroscopic NEC-like lesions. A score of ≥4 in any gut segment was classified as NEC-like. Wet weights of the stomach, small intestine, colon, spleen, kidney and liver were recorded.

### In vitro assessment of gut structure and function

Intestinal morphology, inflammation and mucosal function was assessed postmortem. The paraffin-embedded tissues from the distal part of the small intestine and the colon were sectioned and stained with hematoxylin-eosin and Alcian blue-periodic acid Schiff to measure villous height, crypt depth and goblet cell density, using ImageJ software.^40^ Four digital images per region were obtained at 5 × magnification and used to evaluate the histological structure. To assess intestinal tissue inflammation, interleukin 1-beta (IL-1b) and interleukin 8 (IL-8) levels in the distal small intestine and proximal colon were analysed by porcine-specific enzyme-linked immunosorbent assay (ELISA) as described.^41,42^ The brush-border enzymatic activity was measured in the proximal, middle and distal part of the small intestine with already established methods.^37^ Mucosal enzyme activities were expressed relative to gram wet intestinal tissue and the mean activity level across the three regions was calculated.

From the distal small intestines, total RNA was extracted by homogenising tissue using gentleMACS M-tubes (Miltenyi Biotec) and QIAzol Lysis reagent (Qiagen). RNA was extracted using RNeasy mini kit (Qiagen) treated with RNase-free DNase (Qiagen) according to instructions from the manufacturer. RNA integrity, quantity and purity were measured by Agilent Bioanalyzer (Agilent Technologies) and Nanodrop ND-1000 spectrophotometer (Saveen and Werner AB). A total of 500 ng RNA was used for the cDNA synthesis, and three cDNA replicates were prepared for each RNA sample. Pre-amplification was performed using TaqMan PreAmp Master Mix (Applied Biosystems). A pool of all included primers (200 nM) was prepared and used for pre-amplification as described elsewhere.^43^ After 19 cycles of pre-amplification primer residuals were digested by adding 16 U of Exonuclease I (New England BioLabs) afterwards. Microfluidic high-throughput qPCR was performed on a BioMark real-time instrument (Fluidigm/ Standard BioTools).^44^ Firstly, one 96.96 dynamic array was performed by combining 96 samples with 96 selected primer assays to screen for genes of interest. In a second assay, 50 samples were afterwards analysed in triplicates with 24 primers in a 192.24 dynamic array. See supplementary Table S7 for list of primer names, sequences and lengths of amplicons used. After qPCR, data were curated using Standard BioTools Real-Time PCR Analysis software, followed by data pre-processing in GenEx7 (MultiD Analyses AB), as previously described.^45^ Stable reference genes were determined by GeNorm and NormFinder. Relative gene expression levels were calculated after transforming normalised Cq values to relative quantities scaled to the sample in the data set having the lowest expression of the gene in question.

### Liver clearance capacity

Liver capacity to extract indocyanine green (ICG) was assessed before euthanasia at 24 (one litter) and 72 h (one litter). As ICG is neither metabolised nor reabsorbed by the gut, ICG clearance determines liver function, interacting with portal blood flow. A bolus of 0.5 mg/kg ICG (Verdye, Diagnostic Green, Ascheim-Dornach, Germany) was infused through the umbilical arterial catheter immediately after pre-euthanasia sedation. The ICG clearance was measured spectrophotometrically (LiMON monitor, Pulsion Medical Systems, Munich, Germany), using a finger probe placed on a hind leg, to assess the plasma disappearance rate (PDR). The ratio between ICG concentration immediately and 15 min after ICG infusion (R15) was determined.

### In vivo and in vitro quantification of systemic immunity

Haematological status was evaluated by a basal blood sample taken prior to the in vivo tests and shortly before euthanasia (Fig. 1). Neutrophil phagocytic function was assessed by whole blood incubation with fluorescently labelled *Escherichia coli* for 30 min. Flow cytometry was used to measure the fraction of neutrophils with internalised E. coli, hereby determining the phagocytic rate and capacity as described elsewhere.^46^ Simultaneously, blood was collected for ex vivo stimulation with *Staphylococcus epidermidis* (SE). After blood collection, 300 µL whole blood were incubated for 2 h at 37 °C with 2 × 10^6^ CFU/mL blood as described.^46^ Afterwards, samples were centrifuged at 2000 × *g* for 20 min at 4 °C and plasma was collected for determining levels of tumour necrosis factor α (TNF-α) and interleukin 10 (IL-10) using ELISA.^46^

### Data analysis and statistics

Statistics were performed in R (version 1.4.1103). Continuous data were analysed using a linear mixed-effect model (function lme) including sex and birth weight as fixed effects and litter as random effect. The model assumed normality and homoscedasticity of residuals. Fitted values were assessed, and logarithmic transformation of data was conducted if necessary. If the assumptions of normality and homoscedasticity were not met, a non-parametric test was applied (wilcox.test function). Dichotomous outcomes such as NEC were analysed using a binomial generalised linear model (GLM). Repeated measurements were analysed using repeated measurements analysis of variance (ANOVA). Interaction between the asphyxia intervention and the plasma intervention was tested using ANOVA. Figures were generated using GraphPad software (version 9.4) and data were presented as means ± SD. For comparison of means, *p* < 0.05 was considered a statistically significant difference, while *p* < 0.10 was considered a tendency to an effect.

## Results

### Delivery and neonatal physiological adaptation

**Fig. 2:**
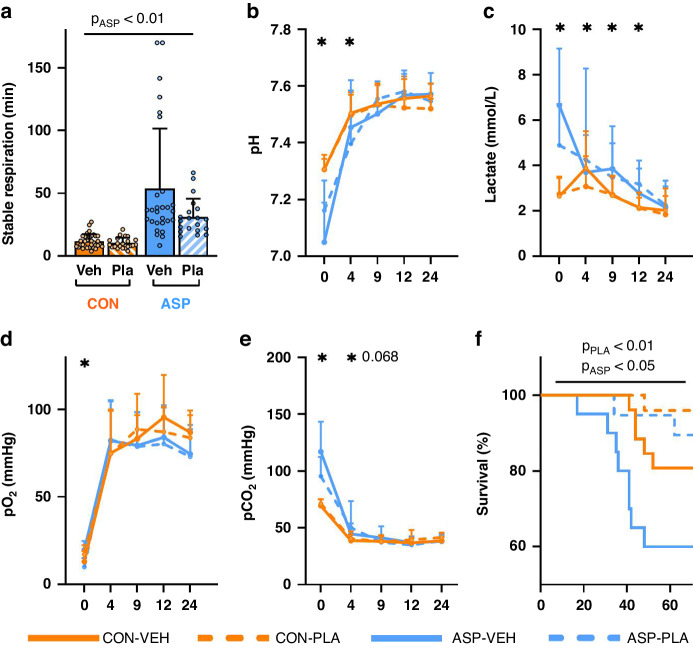
Changes in arterial blood gas values after asphyxic insult in near-term piglets. Time until stable respiration (**a**). Longitudinal data for arterial blood pH (**b**), lactate (**c**), partial pressure of O_2_ (**d**), partial pressure of CO_2_ (**e**) during the first 24 h of life, and survival after successful resuscitation until 72 h of life (**f**). CON control, VEH vehicle, ASP asphyxia, PLA plasma, *P*_ASP_ *p*-value of the main effect of asphyxia relative to control, *P*_PLA_ *p*-value of the main effect of plasma-feeding relative to vehicle. Data are presented as means ± SDs. Asterisks (*) denote *p* < 0.05 of the main effect of asphyxia relative to control at each time point.

Across the five litters of pigs (total *n* = 144), resuscitation failed for 25% of ASP and 3% of CON piglets (*p* < 0.001) in the first few hours after delivery. Time to obtain stable respiration was longer for ASP vs. CON pigs (44.5 ± 38.9 vs. 11.2 ± 5.1 min, *p* < 0.001, Fig. 2). Immediately following cord occlusion, ASP piglets showed increased arterial blood acidity (pH 7.07 ± 0.13 vs. pH 7.31 ± 0.05 in CON pigs), lactate (6.1 ± 2.4 vs. 2.7 ± 0.8 mmol/L) and pCO_2_ levels (110 ± 26 vs. 70 ± 5 mmHg, all *p* < 0.01). The above group differences decreased rapidly in the hours following birth but blood acidity and pCO_2_ levels remained elevated in ASP piglets until 4 h (*p* < 0.05), and lactate levels until 24 h (*p* < 0.05, Fig. 2). One ASP piglet was euthanized due to developing seizures during the study period.

In the study period, the highest mortality was observed for ASP-VEH pigs and lowest for CON-PLA pigs (39%, *n* = 9 vs. 4%, *n* = 1, *p* < 0.05), with intermediate mortality for CON-VEH animals (17%, *n* = 12). No differences in mortality were seen between the two PLA groups (ASP-PLA 10%, *n* = 2 vs. CON-PLA 4%, *n* = 1, *p* > 0.05). Average birth weight was 1179 ± 291 g with no group differences. The two PLA groups had a similar weight gain, while the two VEH groups had lower weight gain or even weight loss (58.0 ± 33.2 vs. −2.9 ± 30.2 g/day, *p* < 0.01). After 24 and 72 h, colon weight relative to body weight was reduced in ASP animals (Table 1, *p* < 0.05), along with a tendency to lower spleen weights at 72 h (*p* = 0.08). Plasma supplementation reduced colon weights and increased intestinal length, compared with VEH treatment (Table 1, both *p* < 0.05), along with a tendency to reduced liver weight (*p* = 0.07).

**Table 1:** Growth and organ weight at 24 h or 72 h of life from plasma- (PLA) or non-plasma-fed (VEH) near term piglets delivered with birth asphyxia (ASP) or without (CON).

Weight	24 h			72 h					
	CON	ASP	*p*_ASP_	CON-VEH	CON-PLA	ASP-VEH	ASP-PLA	*p*_ASP_	*p*_PLA_
Weight gain (g/day)	-	-	-	5.7 ± 30.1	60.7 ± 35.4	−14.5 ± 27.9	61.2 ± 38.6	<0.05	<0.001
Stomach (g)	6.79 ± 0.98	7.24 ± 1.63	ns.	12.06 ± 4.14	11.86 ± 4.84	14.14 ± 5.15	14.15 ± 7.83	ns.	ns.
Stomach content (g)	-	-	-	22.91 ± 15.85	13.30 ± 7.33	15.05 ± 14.34	13.51 ± 6.30	ns.	0.063
SI length (cm)	351 ± 17	343 ± 48	ns.	385 ± 58	363 ± 46	397 ± 59	366 ± 67	ns.	<0.01
SI (g)	41.45 ± 6.03	36.96 ± 10.44	ns.	42.07 ± 10.68	45.29 ± 10.71	44.27 ± 9.97	46.17 ± 11.30	ns.	ns.
Colon (g)	9.80 ± 1.69	8.39 ± 2.19	<0.05	18.98 ± 5.21	16.89 ± 3.85	18.01 ± 4.70	17.17 ± 4.95	<0.05	<0.01
Kidney (g)	11.05 ± 2.14	10.18 ± 2.60	ns.	10.33 ± 3.15	10.11 ± 2.59	11.67 ± 2.78	9.72 ± 2.82	ns.	ns.
Liver (g)	34.28 ± 7.28	33.52 ± 8.86	ns.	35.67 ± 7.57	32.88 ± 7.45	38.30 ± 7.73	35.03 ± 10.32	ns.	0.074
Spleen (g)	-	-	-	2.55 ± 0.79	2.47 ± 0.96	2.35 ± 0.56	2.24 ± 0.63	0.08	ns.

All data is presented as means ± SDs. There was no interaction between the asphyxia and plasma intervention.

*CON* control, *VEH* vehicle, *ASP* asphyxia, *PLA* plasma, *p*_*ASP*_ *p*-value of the effect of asphyxia intervention, *p*_*PLA*_ *p*-value of the effect of plasma-feeding, *SI* small intestine, *ns* not significant.

### Blood biochemistry

At 24 h, ASP animals had lower levels of alkaline phosphatase (Table 2, *p* = 0.051) and gamma-glutamyl transferase (*p* < 0.01), compared with CON animals. Tendencies to higher inorganic phosphorus and cholesterol levels were observed (both *p* < 0.10). After 72 h, ASP animals had lower gamma-glutamyl transferase (Table 2, *p* < 0.05) and higher alanine aminotransferase and creatinine levels (both *p* < 0.05). PLA supplementation increased plasma levels of sodium, iron, cholesterol and creatinine, relative to VEH animals (all *p* < 0.05), together with a tendency to higher magnesium levels (*p* = 0.07). Further, levels of serum amyloid A (an acute phase reactant) were reduced in PLA animals (Table 2, *p* < 0.01), along with reduced calcium, albumin, aspartate aminotransferase, gamma-glutamyl transferase, urea nitrogen and creatinine kinase levels, relative to VEH animals (all *p* < 0.05).

**Table 2:** Blood biochemistry at from the 24 h litter and the 72 h litter from plasma- (PLA) or non-plasma-fed (VEH) near term piglets delivered with birth asphyxia (ASP) or without (CON).

Parameter	24 h			72 h					
	CON (*n* = 10)	ASP (*n* = 11)	*p*_ASP_	CON-VEH (*n* = 25)	CON-PLA (*n* = 25)	ASP-VEH (*n* = 15)	ASP-PLA (*n* = 19)	*p*_ASP_	*p*_PLA_
Serum amyloid A (mg/L)	<5	<5	ns.	25.9 ± 22.0	12.6 ± 8.0	21.1 ± 10.0	11.3 ± 4.2	ns.	<0.01
ALP (U/L)	2177 ± 1117	1325 ± 353	0.051	1141 ± 728	1039 ± 365	1077 ± 601	1259 ± 708	ns.	ns.
ALT (U/L)	18 ± 2	29 ± 24	ns.	14 ± 4	13 ± 3	15 ± 5	16 ± 10	<0.05	ns.
AST (U/L)	74 ± 49	92 ± 69	ns.	58 ± 61	24 ± 25	63 ± 71	28 ± 23	ns.	<0.01
GGT (U/L)	449 ± 88	296 ± 88	<0.01	112 ± 33	84 ± 20	98 ± 36	72 ± 34	0.04	<0.001
Creatine kinase (U/L)	341 ± 250	1382 ± 2275	ns.	139 ± 106	78 ± 98	242 ± 349	92 ± 107	ns.	<0.05
Creatinine (µmol/L)	52 ± 5	65 ± 21	ns.	40 ± 11	49 ± 6	44 ± 15	62 ± 29	<0.01	<0.001
Inorganic P (mmol/L)	1.5 ± 0.2	1.7 ± 0.2	0.071	1.4 ± 0.8	1.3 ± 0.3	1.7 ± 1.1	1.5 ± 0.8	ns.	ns.
Urea nitrogen (mmol/L)	3.7 ± 1.0	3.2 ± 0.8	ns.	6.4 ± 2.0	5.0 ± 2.2	6.8 ± 2.3	5.2 ± 3.9	ns.	<0.05
Cholesterol (mmol/L)	1.14 ± 0.23	1.30 ± 0.19	0.091	1.64 ± 0.32	1.87 ± 0.38	1.77 ± 0.52	1.98 ± 0.42	ns.	<0.01
Total protein (g/L)	26.82 ± 1.57	26.58 ± 1.70	ns.	28.22 ± 3.80	28.32 ± 2.41	27.55 ± 3.79	27.23 ± 3.51	ns.	ns.
Albumin (g/L)	10.54 ± 0.86	10.60 ± 0.91	ns.	13.38 ± 2.21	12.38 ± 1.42	13.51 ± 2.35	12.14 ± 1.88	ns.	<0.05
Iron (µmol/L)	<0.4	<0.4	ns.	5.2 ± 2.4	6.3 ± 2.7	5.1 ± 2.4	5.7 ± 2.1	ns.	<0.05
Magnesium (mmol/L)	<0.21	<0.21	ns.	0.88 ± 0.23	0.82 ± 0.08	0.98 ± 0.31	0.86 ± 0.18	ns.	0.068
Calcium (mmol/L)	<0.25	<0.25	ns.	2.76 ± 0.20	2.67 ± 0.13	2.76 ± 0.25	2.65 ± 0.22	ns.	<0.05
Sodium (mmol/L)	140 ± 3	138 ± 2	ns.	145 ± 5	157 ± 7	143 ± 9	156 ± 5	ns.	<0.001
Potassium (mmol/L)	-	-	-	3.19 ± 1.16	3.04 ± 0.44	3.35 ± 1.14	3.31 ± 1.15	ns.	ns.

All data is presented as means ± SDs. There was no interaction between the asphyxia and plasma intervention.

*CON* control, *VEH* vehicle, *ASP* asphyxia, *PLA* plasma, *p*_*ASP*_, *p*-value for ASP intervention, *p*_*PLA*_ *p*-value for PLA intervention, *U* unit, *ALP* alkaline phosphatase, *ALT* alanine aminotransferase, *AST* aspartate aminotransferase, *GGT* gamma-glutamyl transferase, *ns* not significant.

### Gut transit, permeability and macromolecule absorption and liver function test

**Fig. 3:**
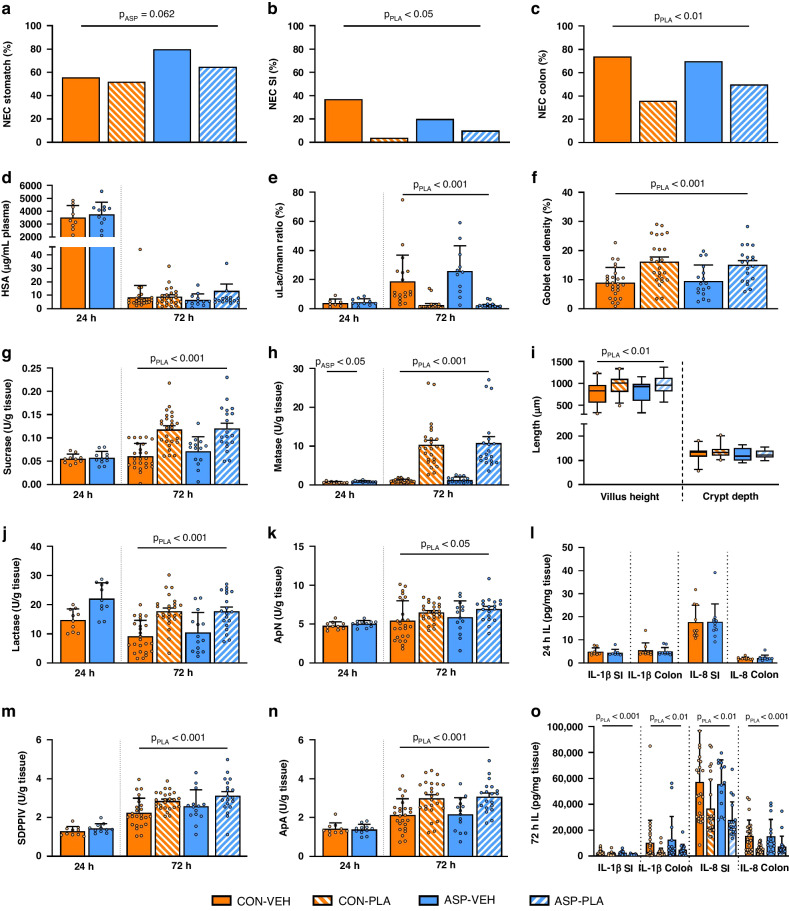
Changes in gut structure and function after aphyxic insult and enteral plasma feeding in near-term piglets. Incidence of NEC-like lesions in the stomach (**a**), small intestine (**b**) and colon (**c**) among four groups of piglets (CON-VEH, CON-PLA, ASP-VEH and ASP-PLA) at 72 h after birth. Intestinal human serum albumin (HSA) absorption at 24 h and 72 h (**d**), urinary lactulose-mannitol ratio indicatory of intestinal permeability (**e**), goblet cell density (**f**), brush-border enzyme activities for disaccharidases (sucrase, maltase, lactase) (**g**, **h**, **j**), and peptidases (ApN aminopeptidase N, DPPIV dipeptidyl peptidase-4, ApA aminopeptidase A) (**k**, **m**, **n**), villus height and crypt depth (**i**), and intestinal tissue interleukin concentration (**l**, **o**) at 24 h and 72 h. CON control, VEH vehicle, ASP asphyxia, PLA plasma, *P*_ASP_ *p*-value of the main effect of asphyxia versus control, *P*_PLA_ *p*-value of the main effect of plasma-feeding versus vehicle, NEC necrotising enterocolitis, SI small intestine, IL interleukin. Data presented as incidences or means ± SDs.

**Fig. 4:**
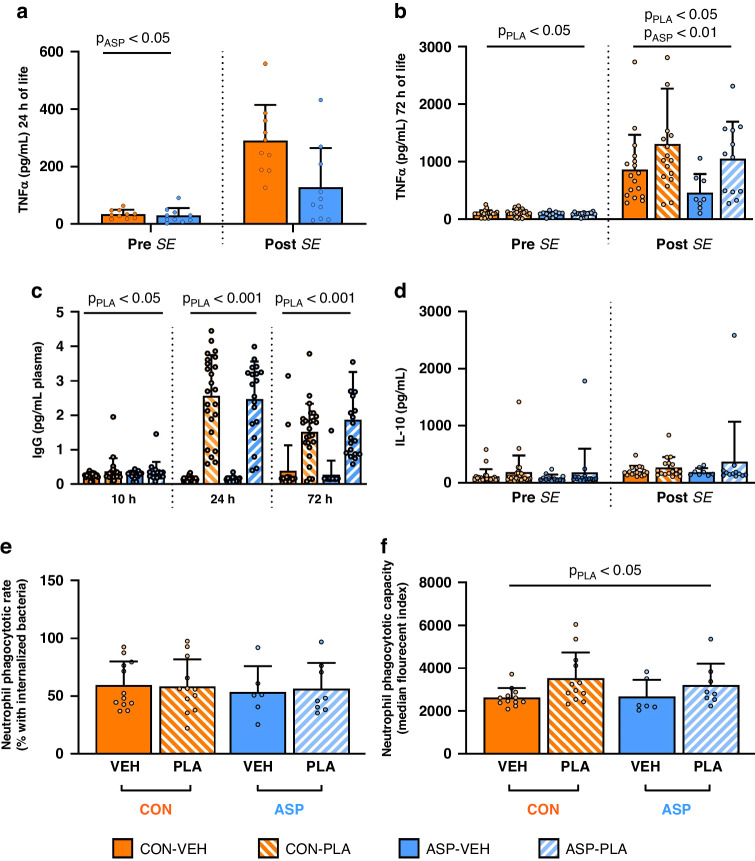
Changes in immunity after asphyxic insult and enteral plasma feeding in near-term piglets. Release of TNF-α cytokine before and after ex vivo whole blood stimulation with *Staphylococcus epidermidis* (SE) at 24 h (**a**) and 72 h of life (**b**), neutrophil phagocytotic rate (**e**) and capacity (**f**) at 72 h of life, along with plasma immunoglobulin concentration at 10–72 h of life (**c**) and IL-10 release before and after *SE* stimulation at 72 h of life (**d**). TNF tumour necrosis factor, *SE* staphylococcus epidermidis, IL interleukin, IgG immunoglobulin G. CON control, VEH vehicle, ASP asphyxia, PLA plasma, *P*_ASP_, *p*-value of the main effect of asphyxia relative to control, *P*_PLA_, *p*-value of the main effect of plasma-feeding versus vehicle. Data presented as means ± SDs.

As measured on day 2, the gut transit time was shorter in both PLA groups compared with VEH groups (CON-PLA 6.1 ± 5.7 and ASP-PLA 5.2 ± 3.6 h vs. CON-VEH 14.4 ± 9.9 and ASP-VEH 7.3 ± 7.9 h, *p* < 0.001). At 24 h, there were no differences in intestinal permeability as determined by the urinary lactulose-mannitol ratio, and the macromolecule endocytotic capacity as determined by the absorption of human serum albumin as a marker (Fig. 3, both *p* > 0.05). At 72 h, gut transit time was reduced in ASP animals (*p* < 0.05), while intestinal permeability was unaffected by ASP (Fig. 3). Intestinal permeability was lower in PLA groups (urinary lactulose-mannitol ratio: CON-PLA 2.7 ± 3.9 and ASP-PLA 2.4 ± 1.9%) compared with the VEH groups (CON-VEH 18.8 ± 18.1 and ASP-VEH 26.0 ± 17.2%, *p* < 0.001). Absorptive capacity for protein macromolecules, as assessed by plasma human serum albumin levels, did not differ between groups (Fig. 3, *p* > 0.05). The plasma levels of IgG were higher from 10 h in both PLA groups, relative to other pigs, and levels remained higher until the end of the study (Fig. 4, all *p* < 0.05).

Regarding liver clearance tested by ICG, neither PDR nor R15 were affected by asphyxia or plasma supplementation (Supplementary Table S6).

### Gut pathology and assessment of gut structure, immunology and function in vitro

Examination after euthanasia at 24 h showed no NEC-like lesions, and the tissue concentrations of IL-1β and IL-8 in the distal part of the small intestine and colon were similar between CON and ASP animals. At 24 h, maltase concentrations in the small intestine were higher in ASP than CON animals (Fig. 3). For the remaining disaccharidases and peptidases, no differences were observed at 24 h (Fig. 3).

At 72 h, there was a tendency to higher NEC incidence in the stomach region of ASP vs. CON animals (Fig. 3, *p* = 0.06), but IL-1β and IL-8 were unaffected by ASP. At 72 h, a reduction in NEC incidences were seen in the small intestine and colon in PLA groups relative to VEH groups (Fig. 3, both *p* < 0.05), along with lower levels of IL-1β and IL-8 in the distal part of the small intestine and colon among PLA animals compared with VEH animals (Fig. 3, all *p* < 0.05). Higher activities of brush-border enzymes were observed in the two PLA groups, relative to VEH groups (Fig. 3, all *p* < 0.05). Likewise, villi heights (distal intestine) and goblet cell densities (colon) were elevated in PLA animals (Fig. 3, all *p* < 0.05).

Whereas the qPCR analyses (supplementary Table S5) showed no effect of ASP relative to CON, PLA animals relative to VEH showed an increase in the relative gene expression of occludin (OCLN, *p* < 0.01) and erb-B2 receptor tyrosine kinase 2 (ERBB2, *p* < 0.01) and a tendency for an increase in tight junction protein 1 (TJP1, *p* = 0.052), hypoxia-inducible factor 1 inhibitor (HIF1AN, *p* = 0.08) and toll-like receptor 4 (TLR4, *p* = 0.1) along with a decrease in IL8 (*p* < 0.01).

### Blood haematology and immune responses

At 24 h, a higher monocyte count was observed in ASP animals compared with CON (Table 3, *p* < 0.05) and a tendency to a higher eosinophil count (*p* = 0.053). At 72 h, these effects of asphyxia on monocyte counts disappeared. Haemoglobin concentration was lower in ASP than CON animals (*p* < 0.05), but reticulocyte and platelet counts did not differ (Table 3). Ex vivo stimulation of whole blood with SE resulted in a lower TNF-α response in the ASP group compared with CON after 24 and 72 h (Fig. 4, both *p* < 0.05). At 72 h, supplementation with plasma increased total leucocyte counts (Table 3, *p* < 0.001), reflecting increases in all leucocyte subtypes (all *p* < 0.01). Ex vivo SE stimulation resulted in higher TNF-α levels in PLA vs. VEH animals (Fig. 4, *p* < 0.05). No group differences were seen in IL-10 levels before or after SE stimulation. Finally, the ex vivo neutrophil phagocytotic capacity toward E. coli where higher in PLA than VEH animals (*p* < 0.05).

**Table 3:** Blood haematology at 24 h or 72 h of life from plasma- (PLA) or non-plasma-fed (VEH) near term piglets delivered with birth asphyxia (ASP) or without (CON).

Parameter	24 h			72 h					
	CON (*n* = 10)	ASP (*n* = 11)	p_ASP_	CON-VEH (*n* = 21)	CON-PLA (*n* = 21)	ASP-VEH (*n* = 13)	ASP-PLA (*n* = 15)	*p*_ASP_	*p*_PLA_
Total leucocytes (10^9^/L)	6.88 ± 1.24	8.51 ± 2.80	ns.	2.95 ± 1.47	4.92 ± 1.86	2.39 ± 1.32	4.87 ± 1.38	ns.	<0.001
Neutrophils (10^9^/L)	5.11 ± 1.01	6.49 ± 2.61	ns.	1.60 ± 1.28	2.90 ± 1.52	1.25 ± 0.97	2.74 ± 1.38	ns.	<0.001
Monocytes (10^9^/L)	0.04 ± 0.01	0.07 ± 0.03	<0.05	0.14 ± 0.10	0.19 ± 0.13	0.12 ± 0.15	0.22 ± 0.09	ns.	<0.01
Eosinophils (10^9^/L)	0.02 ± 0.01	0.02 ± 0.01	0.053^a^	0.01 ± 0.01	0.03 ± 0.03	0.01 ± 0.01	0.02 ± 0.02	ns.	<0.001
Lymphocytes (10^9^/L)	1.70 ± 0.63	1.91 ± 0.78	ns.	1.14 ± 0.38	1.74 ± 0.63	0.96 ± 0.34	1.83 ± 0.51	ns.	<0.001
Total erythrocytes (10^12^/L)	4.43 ± 0.52	3.98 ± 0.46	ns.	3.56 ± 0.54	3.40 ± 0.55	3.14 ± 0.59	3.47 ± 0.46	ns.	ns.
Haemoglobin (mmol/L)	5.7 ± 0.4	5.3 ± 0.7	ns.	4.7 ± 0.7	4.5 ± 0.7	4.2 ± 0.8	4.5 ± 0.5	<0.05	ns.
MCHC (mmol/L)	18.47 ± 0.44	18.84 ± 0.50	ns.	18.62 ± 0.50	17.90 ± 0.58	18.37 ± 0.78	18.14 ± 0.62	ns.	<0.001
MCV (fL)	69.94 ± 3.92	69.98 ± 1.82	ns.	70.91 ± 3.64	73.79 ± 3.15	72.42 ± 3.88	71.01 ± 2.81	ns.	ns.
Reticulocytes (10^9^/L)	324.8 ± 45.3	307.0 ± 56.2	ns.	267.4 ± 86	223.9 ± 58.6	225.4 ± 85.9	270.5 ± 85.8	ns.	ns.
Platelets (10^9^/L)	296 ± 55	319 ± 65	ns.	359 ± 68	304 ± 116	349 ± 121	321 ± 91	ns.	ns.
MPV (fL)	11.1 ± 1.4	10.5 ± 1.0	ns.	11.89 ± 2.52	12.52 ± 2.40	13.17 ± 4.85	12.05 ± 2.47	ns.	ns.

All data is presented as means ± SDs. There was no interaction between the asphyxia and plasma intervention.

*CON* control, *VEH* vehicle, *ASP* asphyxia, *PLA* plasma, *p*_*ASP*_ *p*-value of the effect of asphyxia intervention, *p*_*PLA*_ *p*-value of the effect of plasma-feeding, *MCHC* mean corpuscular haemoglobin concentration, *MCV* mean corpuscular volume, *MPV* mean platelet volume, *ns* not significant.

^a^non-parametric test applied.

## Discussion

Birth asphyxia remains an important clinical problem for both infants and piglets, and it is important to know if compromised umbilical blood flow leads to impaired gut function, interacting with the quality of the first milk diets. Using near-term, caesarean-derived piglets as models for birth-compromised infants and pigs, we found that a short period of birth asphyxia, induced by cord occlusion, did not notably affect gut function. The clinical implication is that newborns successfully resuscitated after a brief period of asphyxia during delivery, do not require special interventions (e.g. antibiotics, diet therapy) to prevent birth-related gut damage or dysfunction during the first days of life. However, both with and without birth asphyxia, enteral supplementation with plasma-induced marked benefits to growth, gut structure, function and immunology in the days after birth. In another study on the same animals, we showed that dietary plasma ameliorated the brain effects of birth-related asphyxia in newborn pigs.^35^ Further studies are required to identify the specific bioactive components in plasma, but our findings suggest that plasma derivatives can be used to develop gut- and brain-protective diet regimens for compromised newborn infants and animals.

Neonatal adaptation was negatively affected by birth-associated cord obstruction with a higher failure of resuscitation, longer time to reach stable respiration and lower blood pH in the first hours after birth. Interestingly, this resulted in high mortality of asphyxiated animals, not only in the first 24 h, but also in the following days. Due to immaturity, even when born close to term, piglets are highly sensitive to neonatal gut and immune dysfunctions, but experimental birth asphyxia did not affect this sensitivity. The tendency to more haemorrhagic NEC-like lesions in the stomach of ASP animals may partly be explained by a longer period of non-invasive ventilation, causing barotraumas to gastric tissue, inducing inflammation and oedema. Birth asphyxia did not affect blood haematology or biochemistry values beyond the immediate neonatal period, indicating that the surviving animals rapidly regained their normal physiology following the birth insult. However, ex vivo stimulation of whole blood with live bacteria led to a lower TNFα response after 24 h. This suggests birth asphyxia negatively affected the inflammatory capacity of immune cells, although the effect was transient and largely disappeared by 72 h. Such depressed immune cell responses are consistent with more cases of sepsis among infants subjected to complicated deliveries.^47^ The lowered haemoglobin levels in ASP piglets at 72 h are not likely an additional asphyxia-related deficit, but rather because umbilical cord milking upon delivery could not be done for ASP piglets (only for CON piglets), thus preventing the final transplacental blood transfusion, as shown for infants.^48^

Plasma supplementation reduced the asphyxia-related mortality (as indicated by similar low mortality for PLA groups) and markedly reduced evidence of gut pathology and dysfunctions at autopsy. The effects were remarkable, considering that all pigs were first fed with bovine colostrum (first 24 h), before transition to formula feeding. We have previously shown that feeding bovine colostrum, before or after formula feeding, protects the immature piglet intestine against formula-induced lesions and dysfunctions.^49,50^ In this study, the additional effect of dietary plasma supplementation was supported by reduced gut permeability, pro-inflammatory cytokines and increased goblet cell density, villous heights and activities of brush-border enzymes in PLA animals. These gut effects were partly connected as villous atrophy is known to reduce activity of peptidases and disaccharidases, and both may affect colonic goblet cell density via altered nutrient digestion and gut microbiota exposure. The benefits of PLA were also confirmed on the transcriptional level by increased expression of TJP1 and OCLN, both important for intestinal barrier along with increased ERBB2, which is important for cell proliferation. In PLA animals, an increase in HIF1AN along with reduced levels of IL8 was also observed, potentially indicative of enhanced anti-inflammatory settings in these subjects. Besides, it is important that plasma supplementation improved the mucosal barrier, potentially preventing adverse immunological responses to colonising bacteria and translocation of bacteria and toxins into the bloodstream. Decreased colon weights reflect reduced oedema and inflammation of colonic tissue.

It is not known which plasma-derived compound(s) mediates the gut-protective effects in this study. Possibly, plasma IgG acts to prevent adverse mucosa-bacteria interactions and reduce local mucosal inflammatory responses, in turn improving structure and digestive functions, as suggested from infant studies on enteral IgA and IgG supplementation.^21,22^ Another large protein component of plasma, albumin, is an important antioxidant and enteral albumin may help to dampen oxidative stress of developing enterocytes, which could be particularly important following ischaemia-reperfusion induced by birth-related cord obstruction. Protective effects of plasma proteins may partly act across species and bovine IgG reduces bacterial translocation in a human intestinal co-culture model,^51^ consistent with the widespread evidence of gut effects of IgG- and albumin-rich bovine colostrum beyond newborn calves alone.^16,19^

Among the affected biochemistry variables, increased levels of sodium and iron by plasma supplementation may be explained by high levels of these minerals in the supplemented porcine plasma, relative to water for VEH animals (Supplementary Table S4), together with less excretion via kidneys and better fluid retention. Conversely, lower albumin, magnesium and calcium levels in PLA pigs may reflect mainly higher total blood volume, but potentially also improved muscle metabolism and growth, in turn increasing creatinine levels and lowering urea levels in plasma-supplemented piglets. The higher daily weight gain among PLA animals may therefore be a combination of genuine tissue growth effects as well as higher fluid retention.

Plasma feeding caused higher levels of all leucocyte subsets along with improved neutrophil phagocytic capacity, probably linked to higher IgG levels in PLA animals. The presence of circulating IgG may improve leucocytes homing—facilitating their release into the blood stream—and their phagocytotic ability, thus increasing phagocytic capacity. It is unlikely that the increased number and function of immune cells was caused by inflammation as serum amyloid A levels were lower in PLA pigs. Likewise, reduced liver weight and enzymes, together with increased cholesterol levels, may reflect improved liver function and dampened systemic (and hepatic) inflammation after dietary plasma supplementation.

Several limitations should be considered. The high mortality among ASP-VEH animals may have led to selection bias since not all prematurely euthanized animals underwent tissue and blood sampling at euthanasia. Low sample size could cause insufficient statistical power for certain endpoints. Although no power calculation was performed, we aimed to have ≥ 15 animals per group based on previous experience.^30^ However, to evaluate the interaction between the asphyxia and plasma intervention, a 4–8 times larger sample size may be needed^52,53^ as insufficient statistical power increases the likelihood of false-negative results (type II error).^54^ Furthermore, CON piglets were mostly delivered before ASP animals, which introduces a risk of prolonged sedation during the caesarean section. Prolonged sedation can increase respiratory depression, extending the time required for stable respiration. Additionally, increased duration spent in utero during the surgical procedure could increase the risk of placental hypoperfusion pre-delivery. However, the arterial blood gas values obtained pre-obstruction in ASP animals did not differ from CON animals and we therefore do not believe that any significant placental hypoperfusion occurred. We acknowledge that an extended exposure to anaesthesia in utero may impact neonatal adaptation, but we consider this as a part of our experimental model. To summarise, the ASP-insult encompasses a three-factor combination: cord obstruction, anaesthesia exposure and pre-delivery time. While this insult was enough to affect some brain outcomes,^35^ the asphyxia did not appear to be severe enough to cause severe intestinal. Yet, the increased mortality of ASP-VEH animals indicates that the chosen insult was appropriate to reflect temporary, birth-related asphyxia in vaginally-delivered piglets or infants.

In conclusion, dietary plasma supplementation improves survival, gut health and ex vivo immune response to bacteria in both control and asphyxiated piglets. Gut health may be improved by anti-inflammation responses counteracting inflammatory insults of formula feeding. The improved ex vivo immune system responses may primarily be caused by increased IgG levels. Further studies are required to determine which components in plasma cause these important protective effects. Finally, it will be critical to know whether such effects depend on associated milk diets (colostrum, formula), time and mode of feeding, preparation of plasma (e.g. freezing, storage, fractionation) and origin of plasma (e.g. mother- and species-specificity of plasma).

## Supplementary information

Supplementary Tables

Supplementary Table S7

## Data Availability

The data generated during and/or analysed during the current study are available from the corresponding author upon request.

## References

[1] Hammad, I. A. et al. Umbilical cord abnormalities and stillbirth. *Obstet. Gynecol.* **135**, 644–652 (2020). 32028503 10.1097/AOG.0000000000003676 PMC7036034

[2] Zahedi-Spung, L. D., Raghuraman, N., Carter, E. B., Cahill, A. G., & Rosenbloom, J. I. Umbilical artery cord gas abnormalities in the presence of a nuchal cord in term singleton pregnancies: a cohort study. *Am. J. Perinatol.* **41**, 853–858 (2024). 10.1055/a-1787-7408 35240709

[3] Młodawska, M., Młodawski, J., Świercz, G. & Zieliński, R. The relationship between nuchal cord and adverse obstetric and neonatal outcomes: retrospective cohort study. *Pediatr. Rep.* **14**, 40–47 (2022). 35225877 10.3390/pediatric14010007 PMC8883893

[4] Koç, E. et al. The effect of asphyxia on gut blood flow in term neonates. *Indian J. Pediatr.* **65**, 297–302 (1998). 10771976 10.1007/BF02752307

[5] Karna, P., Senagore, A. & Chou, C. C. Comparison of the effect of asphyxia, hypoxia, and acidosis on intestinal blood flow and O2 uptake in newborn piglets. *Pediatr. Res.* **20**, 929–932 (1986). 3774406 10.1203/00006450-198610000-00004

[6] Mace, T. P. et al. Effects of severe hypoxemia on mesenteric blood flow in neonatal piglets. *J. Surg. Res.* **80**, 287–294 (1998). 9878326 10.1006/jsre.1998.5451

[7] Ziegler, A. L. et al. Epithelial restitution defect in neonatal jejunum is rescued by juvenile mucosal homogenate in a pig model of intestinal ischemic injury and repair. *PLoS One* **13**, e0200674 (2018). 30138372 10.1371/journal.pone.0200674 PMC6107120

[8] Sun, Z. et al. Phagocytic and intestinal endothelial and epithelial barrier function during the early stage of small intestinal ischemia and reperfusion injury. *Shock* **13**, 209–216 (2000). 10718378 10.1097/00024382-200003000-00007

[9] Sherman, M. P. New concepts of microbial translocation in the neonatal intestine: mechanisms and prevention. *Clin. Perinatol.* **37**, 565–579 (2010). 20813271 10.1016/j.clp.2010.05.006 PMC2933426

[10] Ceran, B., Alyamaç Dizdar, E., Beşer, E., Karaçağlar, N. B., Sarı, F. N. Diagnostic role of systemic inflammatory indices in infants with moderate-to-severe hypoxic ischemic encephalopathy. *Am. J. Perinatol.* **41**, 248–254 (2021). 10.1055/a-1673-1616 34666380

[11] Albertsson, A.-M. et al. The immune response after hypoxia-ischemia in a mouse model of preterm brain injury. *J. Neuroinflammation* **11**, 153 (2014). 25187205 10.1186/s12974-014-0153-z PMC4172879

[12] Reisinger, K. W. et al. Breast-feeding improves gut maturation compared with formula feeding in preterm babies. *J. Pediatr. Gastroenterol. Nutr.* **59**, 720–724 (2014). 25111221 10.1097/MPG.0000000000000523

[13] Hård, A.-L. et al. Review shows that donor milk does not promote the growth and development of preterm infants as well as maternal milk. *Acta Paediatr.* **108**, 998–1007 (2019). 30565323 10.1111/apa.14702 PMC6520191

[14] Huo, M. et al. Intervention effect of oropharyngeal administration of colostrum in preterm infants: a meta-analysis. *Front. Pediatr.* **10**, 895375 (2022). 35832583 10.3389/fped.2022.895375 PMC9271762

[15] Inoue, R. & Tsukahara, T. Composition and physiological functions of the porcine colostrum. *Anim. Sci. J.* **92**, e13618 (2021). 34409709 10.1111/asj.13618 PMC9286568

[16] Sangild, P. T., Vonderohe, C., Melendez Hebib, V. & Burrin, D. G. Potential benefits of bovine colostrum in pediatric nutrition and health. *Nutrients* **13**, 2551 (2021). 34444709 10.3390/nu13082551 PMC8402036

[17] Ren, S. et al. Neonatal gut and immune maturation is determined more by postnatal age than by postconceptional age in moderately preterm pigs. *Am. J. Physiol. Gastrointest. Liver Physiol.* **315**, G855–G867 (2018). 30118350 10.1152/ajpgi.00169.2018

[18] Meier, P., Patel, A. & Esquerra-Zwiers, A. Donor human milk update: evidence, mechanisms, and priorities for research and practice. *J. Pediatr.* **180**, 15–21 (2017). 27773337 10.1016/j.jpeds.2016.09.027 PMC5183469

[19] Ulfman, L. H., Leusen, J. H. W., Savelkoul, H. F. J., Warner, J. O. & van Neerven, R. J. J. Effects of bovine immunoglobulins on immune function, allergy, and infection. *Front. Nutr.* **5**, 52 (2018). 29988421 10.3389/fnut.2018.00052 PMC6024018

[20] Weström, B., Arévalo Sureda, E., Pierzynowska, K., Pierzynowski, S. G. & Pérez-Cano, F.-J. The immature gut barrier and its importance in establishing immunity in newborn mammals. *Front. Immunol.* **11**, 1153 (2020). 32582216 10.3389/fimmu.2020.01153 PMC7296122

[21] Foster, J. P., Seth, R. & Cole, M. J. Oral immunoglobulin for preventing necrotizing enterocolitis in preterm and low birth weight neonates. *Cochrane Database Syst. Rev.* **4**, CD001816 (2016). 27040323 10.1002/14651858.CD001816.pub3 PMC6464658

[22] Eibl, M. M., Wolf, H. M., Fürnkranz, H. & Rosenkranz, A. Prevention of necrotizing enterocolitis in low-birth-weight infants by IgA-IgG feeding. *N. Engl. J. Med.* **319**, 1–7 (1988). 3288866 10.1056/NEJM198807073190101

[23] Czank, C., Prime, D. K., Hartmann, B., Simmer, K. & Hartmann, P. E. Retention of the immunological proteins of pasteurized human milk in relation to pasteurizer design and practice. *Pediatr. Res.* **66**, 374–379 (2009). 19581827 10.1203/PDR.0b013e3181b4554a

[24] Moro, G. E. et al. Processing of donor human milk: update and recommendations from the European Milk Bank Association (EMBA). *Front. Pediatr.* **7**, 49 (2019). 30873395 10.3389/fped.2019.00049 PMC6403467

[25] Pierce, J. L., Cromwell, G. L., Lindemann, M. D., Russell, L. E. & Weaver, E. M. Effects of spray-dried animal plasma and immunoglobulins on performance of early weaned pigs. *J. Anim. Sci.* **83**, 2876–2885 (2005). 16282627 10.2527/2005.83122876x

[26] Balan, P., Staincliffe, M. & Moughan, P. J. Effects of spray-dried animal plasma on the growth performance of weaned piglets—a review. *J. Anim. Physiol. Anim. Nutr.* **105**, 699–714 (2021). 10.1111/jpn.13435 32860645

[27] Jensen, A. R., Elnif, J., Burrin, D. G. & Sangild, P. T. Development of intestinal immunoglobulin absorption and enzyme activities in neonatal pigs is diet dependent. *J. Nutr.* **131**, 3259–3265 (2001). 11739877 10.1093/jn/131.12.3259

[28] Jasion, V. S. & Burnett, B. P. Survival and digestibility of orally-administered immunoglobulin preparations containing IgG through the gastrointestinal tract in humans. *Nutr. J.* **14**, 22 (2015). 25880525 10.1186/s12937-015-0010-7 PMC4355420

[29] Kleist, S. A. & Knoop, K. A. Understanding the elements of maternal protection from systemic bacterial infections during early life. *Nutrients* **12**, 1045 (2020). 32290170 10.3390/nu12041045 PMC7230816

[30] Sangild, P. T. et al. Invited review: the preterm pig as a model in pediatric gastroenterology. *J. Anim. Sci.* **91**, 4713–4729 (2013). 23942716 10.2527/jas.2013-6359 PMC3984402

[31] Sangild, P. T. et al. Diet- and colonization-dependent intestinal dysfunction predisposes to necrotizing enterocolitis in preterm pigs. *Gastroenterology* **130**, 1776–1792 (2006). 16697741 10.1053/j.gastro.2006.02.026

[32] Sun, J. et al. Nutrient fortification of human donor milk affects intestinal function and protein metabolism in preterm pigs. *J. Nutr.* **148**, 336–347 (2018). 29462356 10.1093/jn/nxx033

[33] Yan, X. et al. Bovine colostrum to supplement the first feeding of very preterm infants: The PreColos randomized controlled trial. *Clin. Nutr.* **42**, 1408–1417 (2023). 37437359 10.1016/j.clnu.2023.06.024

[34] Ismail, R. I. H. et al. Gut priming with bovine colostrum and T regulatory cells in preterm neonates: a randomized controlled trial. *Pediatr. Res.* **90**, 650–656 (2021). 33446924 10.1038/s41390-020-01344-y

[35] Ventura, G. C. et al. Enteral plasma supports brain repair in newborn pigs after birth asphyxia. *Brain Behav. Immun.* **119**, 693–708 (2024). 38677626 10.1016/j.bbi.2024.04.032

[36] Sangild, P. T., Ney, D. M., Sigalet, D. L., Vegge, A. & Burrin, D. Animal models of gastrointestinal and liver diseases. Animal models of infant short bowel syndrome: translational relevance and challenges. *Am. J. Physiol. Gastrointest. Liver Physiol.* **307**, G1147–G1168 (2014). 25342047 10.1152/ajpgi.00088.2014 PMC4269678

[37] Østergaard, M. V. et al. Provision of amniotic fluid during parenteral nutrition increases weight gain with limited effects on gut structure, function, immunity, and microbiology in newborn preterm pigs.*JPEN J. Parenter. Enter. Nutr.* **40**, 552–566 (2016). 10.1177/0148607114566463 25613990

[38] Hedegaard, C. J., Lauridsen, C. & Heegaard, P. M. H. H. Purified natural pig immunoglobulins can substitute dietary zinc in reducing piglet post weaning diarrhoea. *Vet. Immunol. Immunopathol.* **186**, 9–14 (2017). 28413052 10.1016/j.vetimm.2017.02.001

[39] Brunse, A., Peng, Y., Li, Y., Lykkesfeldt, J. & Sangild, P. T. Co-bedding of preterm newborn pigs reduces necrotizing enterocolitis incidence independent of vital functions and cortisol levels. *Front. Pediatr.* **9**, 1–11 (2021). 10.3389/fped.2021.636638 PMC8049114 33869114

[40] Schneider, C. A., Rasband, W. S. & Eliceiri, K. W. NIH Image to ImageJ: 25 years of image analysis. *Nat. Methods* **9**, 671–675 (2012). 22930834 10.1038/nmeth.2089 PMC5554542

[41] Østergaard, M. V. et al. Modulation of intestinal inflammation by minimal enteral nutrition with amniotic fluid in preterm pigs. *JPEN J. Parenter. Enter. Nutr.* **38**, 576–586 (2014). 10.1177/0148607113489313 23715776

[42] Van Haver, E. R. et al. Diet-dependent mucosal colonization and interleukin-1beta responses in preterm pigs susceptible to necrotizing enterocolitis. *J. Pediatr. Gastroenterol. Nutr.* **49**, 90–98 (2009). 19516189 10.1097/MPG.0b013e31818de393

[43] Starbæk, S. M. R. et al. Innate antiviral responses in porcine nasal mucosal explants inoculated with influenza A virus are comparable with responses in respiratory tissues after viral infection. *Immunobiology* **227**, 152192 (2022). 35255458 10.1016/j.imbio.2022.152192 PMC8863374

[44] Brogaard, L. et al. IFN-λ and microRNAs are important modulators of the pulmonary innate immune response against influenza A (H1N2) infection in pigs. *PLoS One* **13**, e0194765 (2018). 29677213 10.1371/journal.pone.0194765 PMC5909910

[45] Barington, K., Skovgaard, K., Henriksen, N. L., Johansen, A. S. B. & Jensen, H. E. The intensity of the inflammatory response in experimental porcine bruises depends on time, anatomical location and sampling site. *J. Forensic Leg. Med.* **58**, 130–139 (2018). 29966813 10.1016/j.jflm.2018.06.005

[46] Bæk, O. et al. Infant formula based on milk fat affects immune development in both normal birthweight and fetal growth restricted neonatal piglets. *Nutrients* **13**, 3310 (2021). 34684311 10.3390/nu13103310 PMC8539276

[47] Stafford, I. A. et al. The strong correlation between neonatal early-onset Group B Streptococcal disease and necrotizing enterocolitis. *Eur. J. Obstet. Gynecol. Reprod. Biol.* **223**, 93–97 (2018). 29501938 10.1016/j.ejogrb.2018.02.024

[48] Hosono, S. et al. Umbilical cord milking reduces the need for red cell transfusions and improves neonatal adaptation in infants born at less than 29 weeks’ gestation: a randomised controlled trial. *Arch. Dis. Child Fetal Neonatal Ed.* **93**, F14–F19 (2008). 17234653 10.1136/adc.2006.108902

[49] Li, Y. et al. Bovine colostrum before or after formula feeding improves systemic immune protection and gut function in newborn preterm pigs. *Front. Immunol.* **10**, 3062 (2019). 32082298 10.3389/fimmu.2019.03062 PMC7002359

[50] Yan, X. et al. Supplementary bovine colostrum feedings to formula-fed preterm pigs improve gut function and reduce necrotizing enterocolitis. *J. Pediatr. Gastroenterol. Nutr.* **73**, e39–e46 (2021). 33853107 10.1097/MPG.0000000000003147

[51] Detzel, C. J. et al. Bovine immunoglobulin/protein isolate binds pro-inflammatory bacterial compounds and prevents immune activation in an intestinal co-culture model. *PLoS One* **10**, e0120278 (2015). 25830826 10.1371/journal.pone.0120278 PMC4382133

[52] Fleiss, J. L. *The Design and Analysis of Clinical Experiments* (Wiley, 2011).

[53] Leon, A. C. & Heo, M. Sample sizes required to detect interactions between two binary fixed-effects in a mixed-effects linear regression model. *Comput. Stat. Data Anal.* **53**, 603–608 (2009). 20084090 10.1016/j.csda.2008.06.010 PMC2678722

[54] Akobeng, A. K. Understanding type I and type II errors, statistical power and sample size. *Acta Paediatr.* **105**, 605–609 (2016). 26935977 10.1111/apa.13384

