# Supplementary material for: Enteral plasma feeding improves gut function and immunity in piglets after birth asphyxia
Source: Pediatr Res. 2024 Jul 21;97(2):774–84. doi: 10.1038/s41390-024-03376-0 (PMC12014487; doi:10.1038/s41390-024-03376-0)
Supplement: Supplementary file 1 — Supplementary Tables [file 41390_2024_3376_MOESM1_ESM.pdf]

Supplementary Table S1. Overview of total number of piglets born by cesarean section and resuscitated as either controls (CON) or with induced asphyxia (ASP), and number of piglets euthanized prematurely during the experiment related to congenital, NEC or iatrogenic reasons. The table further indicates how many blood and tissue samples were collected from animals requiring premature euthanasia, and from animals with planned tissue collection at 72h after birth.

	CON	ASP
Total number of pigs delivered	57	57
Successful resuscitation	57	45
Euthanized at birth	2[a]	1[b]

		CON-PLA (n=26)	CON-VEH (n=29)	ASP-PLA (n=21)	ASP-VEH (n=23)
Day 1	Euthanized	n=1[c]	n=2[c]	n=1[c]	n=2[c]
Day 2	Euthanized			n=1[d]	n=4[d]
	NEC scored			n=1	n=4
	Intestine sampled			n=1	n=0
	Blood sampled			n=1	n=0
	Organ sampled			n=1	n=0
Day 3	Euthanized	n=1[d]	n=5[d]	n=1[c]	n=3[d]
	NEC scored	n=1	n=5	n=0	n=3
	Intestine sampled	n=1	n=3	n=0	n=1
	Blood sampled	n=1	n=4	n=1	n=3
	Organ sampled	n=1	n=5	n=1	n=3
Day 4	Euthanized			n=1[d]	n=1[c] and n=2[d]
	NEC scored			n=1	n=3
	Intestine sampled			n=0	n=1
	Blood sampled			n=0	n=2
	Organ sampled			n=1	n=2
Tissue collected at 72 h		n=24	n=21[e]	n=17	n=11

[a]euthanized due to congenital skin defects; [b]euthanized due to excessive bleeding; [c]euthanized due to iatrogenic causes; [d]euthanized due to NEC-like clinical condition; [e]one animal excluded from further analysis.

Supplementary Table S2. Enteral diet composition for colostrum-based diet fed <24 after birth, and milk-replacer based diet fed 24-72h after birth

	**<u><24 h after birth</u>**		**<u>24-72h after birth</u>**	
	**PLA**	**VEH**	**PLA**	**VEH**
Lactose powder [a], g/L			10	10
MCT emulsion [b], g/L			70	70
LCT emulsion [c], g/L			20	20
Maltodextrin powder [d], g/L			30	30
Whey protein powder [e], g/L		70	30	105
Vitamin and mineral mix [f], g/L	2	2	2	2
Bovine colostrum powder [g], g/L	100	100		
Sow porcine plasma, ml	1000		955	
Water, ml		1000		955
Energy, kJ/L	3365	3343	3866	3824
Protein, g/L	130	130	94	93
Whey protein, g/L	43	113	27	93
Carbohydrate, g/L	10	10	37	37
Sugars, g/L	10	10	11	11
Fat, g/L	27	27	45	45
Sat. fat, g/L			34	34
Mono-unsat. fat, g/L			6	6
Poly-unsat. fat, g/L			3	3
MCT, g/L			35	35
LCT, g/L			10	10
Na, mg/L	3320	522	3375	696
K, mg/L	172	1044	557	1372
Ca, mg/L	91	132	126	164
P, mg/L	86	160	144	212
Mg, mg/L	19	25	18	24
Fe, mg/L	921	0	880	0

[a]Variolac 855, Arla Foods Ingredients, Viby J, Denmark; [b]Liquigen MCT, [c]Calogen LCT, and [d]Fantomalt, Nutricia Allerod, Denmark, [e]DI-9224/WPC/WOI 90, Arla Foods Ingredients, [f]Phlexy Vits, Nutricia, [g]ColoDan, Biofiber-Damino, Gesten, Denmark.

Supplementary Table S3. Enteral feeding volumes and parenteral infusion rates.

**Day**	**1**	**2**	**3**	**4**
EN, mL/kg/3 h (mL/kg/day)	15 (120)	15 (120)	15 (120)	15 (120)
PN, mL/kg/h (mL/kg/day)	2 (48)	2 (48)	2 (48)	2 (48)
Total fluid mL/kg/day	168	168	168	168

EN, enteral nutrition; PN, parenteral nutrition. Note: litter 2 only received 42 mL/kg/day of PN and a total of 140mL/kg/day of fluid.

Supplementary Table S4. Biochemistry data for porcine plasma and tap water used as fluid phase for powdered colostrum (first 24 h) and formula (day 2-4).

**Parameters**	**Tap water***	**Porcine plasma (n=3)**
Serum amyloid A (mg/L)	0	0
ALP (U/L)	0	39 ± 6
ALT (U/L)	0	34 ± 12
AST (U/L)	0	16 ± 2
GGT (U/L)	0	40 ± 2
Creatine kinase (U/L)	0	691 ± 149
Enzymatic creatine (umol/L)	0	230 ± 26
Inorganic phosphorus (mmol/L)	0	2.8 ± 0.2
Urea nitrogen (mmol/L)	0	3.8 ± 0.5
Cholesterol (mmol/L)	0	1.35 ± 0.13
Total protein (g/L)	0	57.5 ± 0.4
Albumin (g/L)	0	32.35 ± 2.91
Iron (umol/L)	0.3	16.5 ± 1.4
Magnesium (mmol/L)	1.03	0.80 ± 0.07
Calcium (mmol/L)	1.3	2.26 ± 0.07
Sodium (mmol/L)	5.3	144.4 ± 0.6
Potassium (mmol/L)	0.11	4.39 ± 0.29

U, unit; ALP; alkaline phosphatase; ALT, alanine aminotransferase; AST, aspartate aminotransferase; GGT, gamma-glutamyl transferase; ns, not significant. All data presented as means ± SDs. *Data obtained from local water supply authority (Frederiksberg Grundvand).

Supplementary Table S5. Differential expression of genes at 72 h of life from plasma- (PLA) or non-plasma-fed (VEH) near term piglets delivered with birth asphyxia (ASP) or without (CON).

Gene	CON-VEH (n = 12)	CON-PLA (n = 15)	ASP-VEH (n = 7)	ASP-PLA (n = 14)	p_{ASP}	p_{PLA}
SAA	522 ± 336	368 ± 373	580 ± 378	461 ± 326	ns	ns
TNFα	4.7 ± 3.2	4.0 ± 2.4	3.9 ± 1.9	4.4 ± 2.7	ns	ns
MUC1	3.7 ± 1.8	3.9 ±1.6	4.3 ± 2.5	3.4 ± 1.7	ns	ns
MUC2	4.3 ± 1.5	4.9 ± 3.4	4.1 ± 2.5	4.3 ± 2.5	ns	ns
MYD88	2.6 ± 0.5	3.1 ± 1.1	2.7 ± 0.6	2.9 ± 0.7	ns	ns
TLR4	3.6 ± 1.3	4.3 ± 2.0	3.6 ± 1.8	4.4 ± 1.0	ns	0.095
NOS2	19.7 ± 22.4	39.4 ± 84.5	24.6 ± 15.5	14.9 ± 20.6	ns	ns
VCAM1	8.0 ± 6.4	5.4 ± 3.5	6.6 ± 4.3	8.1 ± 6.3	ns	ns
OCLN	1.7 ± 0.5	2.8 ± 1.3	2.1 ± 0.7	2.4 ± 0.7	ns	< 0.01
CASP3	2.1 ± 0.7	2.3 ± 1.0	2.0 ± 0.6	2.1 ± 1.1	ns	ns
ERBB2	3.0 ± 1.3	4.3 ±1.8	3.7 ± 1.8	4.9 ± 1.9	ns	< 0.01
TJP1	1.5 ± 0.4	1.9 ± 0.9	1.7 ± 0.7	1.9 ± 0.5	ns	0.052
HIF1A	1.4 ± 0.3	1.6 ± 0.5	1.4 ± 0.3	1.4 ± 0.3	ns	ns
VEGFA	1.8 ± 0.7	2.3 ± 1.0	2.0 ± 0.9	1.9 ± 0.5	ns	ns
FGF2	3.1 ± 1.1	3.3 ± 1.4	3.5 ± 1.9	3.8 ± 2.0	ns	ns
SOD2	2.1 ± 1.1	2.2 ± 1.0	2.0 ± 0.6	1.7 ± 0.5	ns	ns
LGALS3	6.4 ± 2.5	9.6 ± 4.8	7.2 ± 2.7	8.6 ± 4.2	ns	ns
IL8	8.6 ± 8.5	4.9 ± 2.7	11.1 ± 6.7	5.0 ± 7.0	ns	< 0.01
HIF1AN	1.6 ± 0.3	1.8 ± 0.4	1.6 ± 0.5	1.7 ± 0.3	ns	0.084
FABP2	73 ± 41	77 ± 57	47 ± 11	117 ± 123	ns	ns

List of gene names see supplementary table S5. CON, control; VEH, vehicle; ASP, asphyxia; PLA, plasma; p_{ASP}, p-value of the effect of asphyxia intervention; p_{PLA}, p-value of the effect of plasma-feeding; ns, not significant. All data is presented as means relative expressions ± SDs. There was no interaction between the asphyxia and plasma intervention.

Supplementary Table S6. Indocyanine green clearance at 24 h or 72 h of life in piglets supplemented with plasma (PLA) or vehicle (VEH) delivered with birth asphyxia (ASP) or without (CON).

	**24 h**			**72 h**					
	**CON**	**ASP**	**p_{ASP}**	**CON**	**ASP**	**VEH**	**PLA**	**p_{ASP}**	**p_{PLA}**
n	10	11		10	5	7	8		
PDR (%/min)	12.49 ±1.36	11.77 ±2.15	ns.	11.64 ±2.07	11.88 ±2.30	11.45 ±1.87	11.95 ±2.33	ns.	ns.
R15 (%)	15.77 ±2.68	17.89 ±5.79	ns.	18.28 ±6.40	17.66 ±6.38	18.53 ±5.18	17.68 ±7.26	ns.	ns.

CON, control; VEH, vehicle; ASP, asphyxia; PLA, plasma; n, number of individuals by combining e.g. the two PLA groups at 72 h; p_{ASP}, p-value of the effect of asphyxia intervention; p_{PLA}, p-value of the effect of plasma-feeding; PDR: Plasma disappearance rate of indocyanine green, R15: retention ratio between plasma concentration of indocyanine green 15 min after infusion and the concentration right after infusion, ns: not significant. All data are presented as means ± SD.
